# Optimising Ambient Setting Bayer Derived Fly Ash Geopolymers

**DOI:** 10.3390/ma9050392

**Published:** 2016-05-19

**Authors:** Evan Jamieson, Catherine S. Kealley, Arie van Riessen, Robert D. Hart

**Affiliations:** 1 School of Civil and Mechanical Engineering, Curtin University, GPO Box U1987, Perth, WA 6845, Australia; evan.jamieson@curtin.edu.au; 2 Technology Delivery Group, Alcoa World Alumina, P.O. Box 161, Kwinana, WA 6966, Australia; 3 John de Laeter Centre, Curtin University, GPO Box U1987, Perth, WA 6845, Australia; Cat.kealley@curtin.edu.au (C.S.K.)

**Keywords:** Bayer liquor, geopolymer, fly ash, ambient curing

## Abstract

The Bayer process utilises high concentrations of caustic and elevated temperature to liberate alumina from bauxite, for the production of aluminium and other chemicals. Within Australia, this process results in 40 million tonnes of mineral residues (Red mud) each year. Over the same period, the energy production sector will produce 14 million tonnes of coal combustion products (Fly ash). Both industrial residues require impoundment storage, yet combining some of these components can produce geopolymers, an alternative to cement. Geopolymers derived from Bayer liquor and fly ash have been made successfully with a compressive strength in excess of 40 MPa after oven curing. However, any product from these industries would require large volume applications with robust operational conditions to maximise utilisation. To facilitate potential unconfined large-scale production, Bayer derived fly ash geopolymers have been optimised to achieve ambient curing. Fly ash from two different power stations have been successfully trialled showing the versatility of the Bayer liquor-ash combination for making geopolymers.

## 1. Introduction

Aluminosilicate inorganic polymers or geopolymers are amorphous materials that have the potential to be an alternative for Ordinary Portland Cement (OPC) binders. Geopolymers have high compressive and flexural strength, high temperature resistance, and impressive acid resistance [1,2,3,4]. Another potential benefit of geopolymers is the significant reduction in CO_2_ emissions. Geopolymer production has been reported to create as little as 6% to 64% of the CO_2_ output compared to OPC production, depending on assessment methodology and lifecycle analysis [5,6,7,8]. Geopolymers have also been shown to immobilise waste within their three-dimensional structure [9,10]. Geopolymers have been considered as an alternative for OPC in applications such as cement pathways, pavers, mine backfill, railway sleepers (ties), sewerage pipes and earth retaining walls [11,12,13].

Geopolymers can be produced from a range of aluminate and silicate materials including metakaolin, fly ash, ground granulated blast furnace slags and mineral processing wastes. As such, most industrial precincts would produce a range of suitable feedstock to enable geopolymer production [14]. Other materials required for concrete production such as residue sand have been evaluated for construction purposes [15,16,17,18]. The use of multiple and varied geopolymer feedstock has led to a focus on understanding the chemistry of the amorphous reactive components. This in turn allows geopolymer products to be formulated with predictable performance properties, without trial and error [14,19,20,21,22]. The critical feedstock for geopolymers include: (i) caustic; (ii) soluble silica; and (iii) soluble alumina. All three materials must be available in large quantities and at appropriate cost for commercial application. In addition, sources of heat may be required as geopolymers are commonly cured at slightly elevated temperatures (60–90 °C). Thermal curing may be suitable for many product applications, but it is impractical in high volume markets such as *in situ* poured paths, roads and curbing. It is possible to design formulations for low calcium-based precursors that can be cured at ambient temperatures by the addition of calcium-containing compounds such as lime or ground granulated blast furnace slag [1,2,23,24,25,26,27,28].

Typical preparation of a geopolymer binder requires a highly concentrated caustic silicate solution to be added to a source of soluble silica and alumina (usually fly ash, metakaolin or ground granulated blast furnace slag) [27]. This procedure commonly targets a reactive Si/Al ratio of around two. Geopolymers may also be made by adding caustic sodium aluminate solution to sources of soluble silica [6,29,30,31,32]. Further development has shown that geopolymers can be made from silica rich, class F fly ash and alumina and caustic rich spent Bayer liquors (A “Bayer liquor” is a process solution derived from the hydrometallurgical thermal caustic extraction of alumina from bauxite ore) [10]. This achieves industrial synergies removing impurity laden liquor from Bayer process streams while providing an inexpensive source of caustic for the production of geopolymer concrete [33,34,35]. There are associated significant reductions in embodied energy of the resultant geopolymer [6].

For geopolymers to be economically viable, large volume production of products are required along with product acceptance [35,36,37]. To this aim, a variety of construction materials are envisaged, many requiring *in situ* and ambient temperature curing. This paper seeks to demonstrate ambient temperature curing of Bayer derived fly ash geopolymers as an enabler to further development. The present work has targeted compressive strength above 20 MPa, as this is typically specified for applications such as pathways, driveways and building footings (AS3727, 1993) [38].

## 2. Experimental

### 2.1. Materials

Bayer plant liquor was from Alcoa of Australia’s Kwinana or Wagerup refineries. Spent liquor (post precipitation), was evaporated to provide the correct H_2_O to Na_2_O ratios in the final product. Refinery liquors are not constant and can vary between locations, highlighting the necessity of fundamental chemical analysis to maintain geopolymer ratios. Table 1 shows the analyses of the two liquors used in this project. Class F fly ash, used as a source of reactive silica and additional alumina, was obtained from Muja or Collie Power Stations, Western Australia. The total elemental composition is presented in Table 2, the phase composition in Table 3, the reactive components of the fly ash are given in Table 4 and particle sizes are provided in Table 5. To achieve specific Si/Al ratios, silica fume (94% purity) obtained from Doral Fused Materials Pty Ltd. (Rockingham, Western Australia, Australia) was added, as required. Particle size is included in Table 5.

Calcium additives for promotion of ambient curing were ground granulated blast furnace slag (Builders Choice, BFS20, BGC Cement, Canning Vale, Western Australia, Australia) and Hylime (Cockburn Cement, Munster, Australia), a manufactured hydrated lime product, predominantly Portlandite (Ca(OH)_2_), that incorporates a small amount of air-entraining agent to enhance water retention and improve plasticity in mortars. Hylime contained 93 wt % Ca(OH)_2_ as Portlandite, and the ground granulated blast furnace slag contained approximately 43 wt % of CaO.

### 2.2. Synthesis

Geopolymer binder compositions were selected to target Si/Al ratios of between 1.0 and 2.4 while keeping the OH/Al ratio close to 0.8 or lower. Unless stated elsewhere in the text, the standard geopolymer binder composition utilised was Si/Al = 2.3 and OH/Al = 0.8.

The dry powders were mixed together, and then Bayer liquor and water were added by mixing for a period of ten minutes before being placed into 25 mm diameter vials. Each sample was prodded with a rod during loading in order to remove air bubbles. Samples were sealed then cured at either 20 °C, 60 °C, 70 °C, 80 °C or 90 °C for 24 h, then left at room temperature for seven days prior to compressive strength testing.

### 2.3. Characterisation

The composition of the reactive amorphous component of the fly ash was determined by subtracting the crystalline composition of the fly ash determined by quantitative the XRD phase analysis from the bulk chemical composition total analysis determined by XRF, as described elsewhere [19].

Compressive strength tests were carried out in accordance with ASTM C39 [39] using a Lloyds Instruments 6000R compressive/tensile strength machine (Bognor Regis, UK) fitted with a 50 kN load cell. A minimum of six samples (25 mm diameter and 50 mm length) from each formulation were tested and the mean and standard deviations reported.

Polished samples for SEM/EDS analysis were mounted on an aluminium stub using carbon tape and then coated with carbon or platinum and observed using a Zeiss NEON 40 EsB scanning electron microscope (Oberkochen, Germany). The microscope was coupled with an Oxford Instruments Energy Dispersive X-ray Spectrometer (High Wycombe, UK) for elemental analysis. Analysis of collected spectra was carried out using Oxford Inca software (High Wycombe, UK).

## 3. Results

### 3.1. Curing Temperature

**Figure 1:**
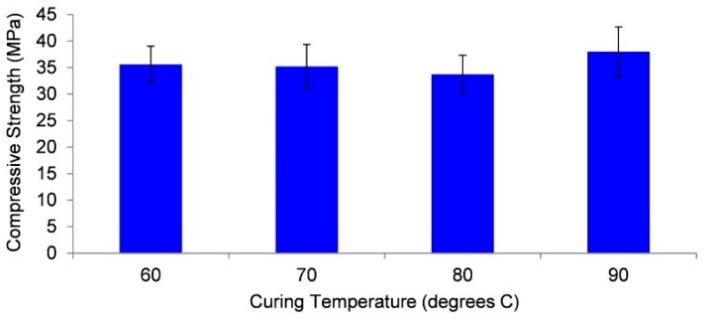
Seven-day compressive strength results from oven cured Collie fly ash/Bayer liquor geopolymer samples.

Sealed samples of Collie fly ash/Bayer liquor geopolymer were cured for 24 h from 60 to 90 °C followed by resting for six days at ambient temperature. Different curing temperatures of sealed samples appear to have little impact upon compressive strength as shown in Figure 1.

Figure 1 shows that the compressive strength after seven days of Collie fly ash/Bayer liquor geopolymer remains consistent at approximately 35 MPa over a curing (24 h) temperature range of 60 to 90 °C. The ability to maintain constant strength with variable curing temperature is in contrast to others who have found increased curing temperatures above 60 °C, resulting in lower compressive strength [40]. This difference may be a result of the source of fly ash but could be due to the small sample volume and the sealed curing environment. The advantage is sufficient to warrant further investigation on larger scale samples.

### 3.2. Silica Fume Content

**Figure 2:**
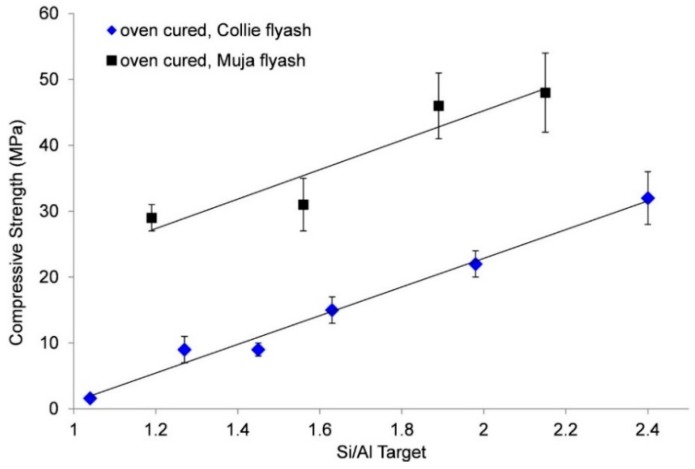
Silica fume addition to Muja fly ash/Bayer liquor geopolymers. Change in compressive strength *versus* targeted Si/Al for oven cured samples.

Silica fume was added to geopolymer paste mixes to achieve targeted Si/Al ratios. The addition of silica fume reduced the workability of the mixture. Geopolymer samples made from Collie or Muja fly ash were oven cured at 70 °C for 24 h in a sealed vial. Figure 2 shows a linear relationship between the targeted Si/Al ratio and the compressive strength.

From Figure 2, it is clear that increasing the Si/Al ratio increases the compressive strength for both sources of fly ash. However, it is not possible to ascertain if the increased strength comes from increased geopolymer gel formation or a separate silica rich phase.

**Figure 3:**
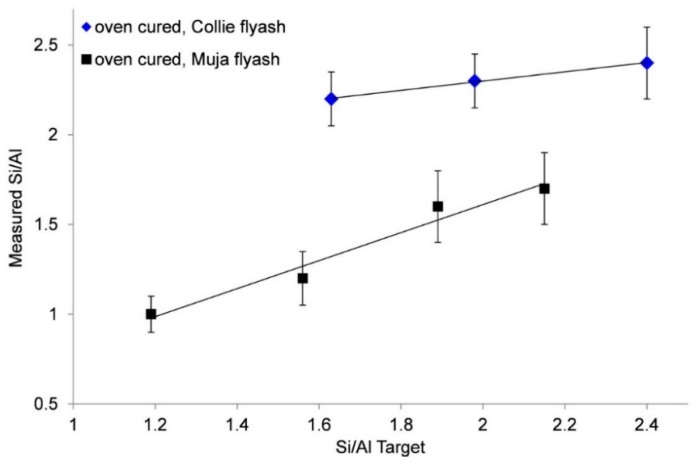
Variation in targeted Si/Al *versus* measured Si/Al of oven cured Collie and Muja fly ash/Bayer liquor geopolymers.

To determine the role of the silica fume, the Si/Al of the geopolymer gel was measured by SEM/EDS. The measured Si/Al ratio was then compared to the targeted ratio. For Collie fly ash geopolymers, the targeted Si/Al and measured Si/Al (EDS) are similar above ratio 1.6 (Figure 3) and are identical at Si/Al of 2.4 This suggests that all of the reactive Si and Al in the fly ash, plus all of the silica fume, has been consumed.

Comparison of the targeted Si/Al with measured Si/Al for the Muja fly ash geopolymer (Figure 3) showed that the measured values were below the targeted values, indicating that not all of the extra Si from the silica fume is being incorporated into the geopolymer. It is thought that the smaller particle size distribution of the Muja fly ash and higher reactivity may result in less added silica being utilised. It is envisaged that if the fly ash dissolves faster than the silica fume, then the geopolymer condenses with a lower than expected Si/Al leaving excess silica fume in the structure.

**Figure 4:**
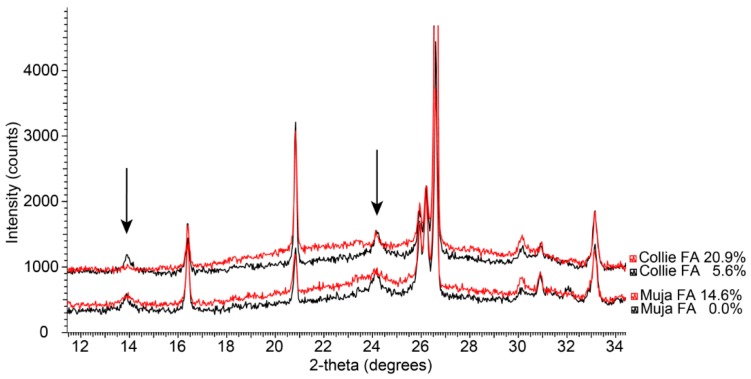
A comparison of the XRD patterns (11°–35° 2θ) collected from Collie and Muja fly ash geopolymers with varying levels of silica fume. Note: The patterns have been offset vertically to improve clarity. The oven-cured samples were cured for 24 h at 70 °C. The most intense reflections of the sodalite phase are highlighted with arrows.

Analysis by XRD (Figure 4) has shown the presence of zeolite (sodalite) with peak intensity higher at lower Si/Al ratios. It is not clear if the presence of sodalite reduces the strength of the geopolymer, or if it is an artefact of incomplete geopolymerisation.

For the oven-cured geopolymer pastes, both Collie and Muja fly ash based samples (Figure 2) showed an increase in the compressive strength with increased silica fume content. However, Muja fly ash geopolymer with a low Si/Al = 1.19 (no added silica fume) still had a compressive strength of 29 MPa, well above the target of 20 MPa.

### 3.3. Ambient Curing

For many high volume premixed geopolymer applications, it is not feasible to cure at elevated temperatures. Thus, ambient curing is required. The incorporation of Ca(OH)_2_ or ground granulated blast furnace slag into the geopolymer mixture facilitates ambient curing. Ground granulated blast furnace slag is significantly less expensive than Ca(OH)_2_; however, larger quantities are required to achieve a compressive strength of 20 MPa.

**Figure 5:**
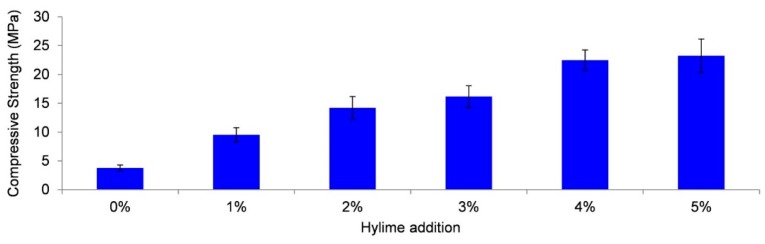
Seven-day compressive strength results for ambient cured Collie fly ash/Bayer liquor geopolymer samples with increasing Ca(OH)_2_ (Hylime) content (wt %).

Samples of Collie fly ash/Bayer liquor geopolymer were produced with different levels of Hylime (Ca(OH)_2_). Figure 5 shows the seven-day compressive strength for samples cured at ambient temperature (approximately 22 °C). The strength increases with increasing Ca(OH)_2_ content with mixtures of 4 wt % or higher Ca(OH)_2_ achieving the desired level (>20 MPa).

**Figure 6:**
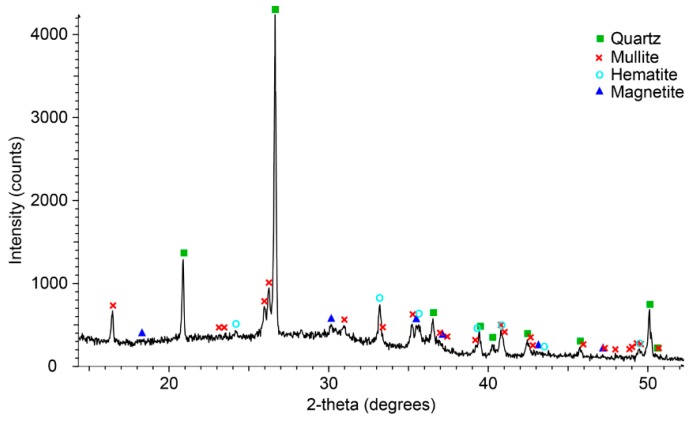
XRD pattern showing phase identification of the Collie fly ash/Bayer liquor based geopolymer paste with 5 wt % Ca(OH)_2_ (Hylime).

Phase identification with XRD data (Figure 6) shows that even at the highest concentrations (5 wt % of Ca(OH)_2_), Portlandite is not present as a separate crystalline phase but is incorporated in the geopolymer structure. Phases identified in the geopolymer paste are quartz, mullite, hematite and magnetite. There was an absence of sodalite compared to the oven cured samples in Figure 4.

**Figure 7:**
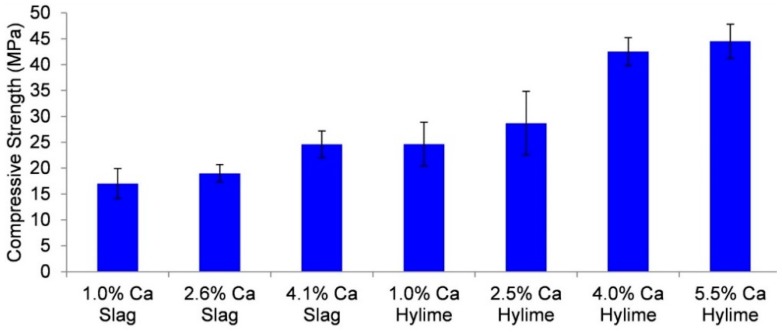
Muja fly ash/Bayer liquor—seven-day ambient cure compressive strength for calcium from different sources.

Ground granulated blast furnace slag (Builders slag) is an industrial residue that can be used as a source of calcium (42.9 wt % CaO). Samples of Muja fly ash/Bayer liquor geopolymer were produced utilising ground granulated blast furnace slag or Hylime for direct comparison. Figure 7 shows the comparative compressive strengths after seven days of ambient cure.

For similar calcium content, it is evident that Hylime (Ca(OH)_2_) is more effective at strength generation. It is understood that the particle size of the ground granulated blast furnace slag will dictate how readily the calcium is provided for the geopolymerisation process. Having a large impact upon strength, it would appear essential for any production process to ensure strict quality control/quality assurance upon the calcium delivery reagent.

The results demonstrate that Muja fly ash derived geopolymer (Figure 7) has higher compressive strength compared to Collie fly ash derived geopolymer (Figure 5). The Muja product also requires less calcium to achieve the ambient cure compressive strengths target. It is believed that the higher surface area of Muja ash (Table 5) contributed to improved alkali reactivity leading to greater geopolymer formation.

**Figure 8:**
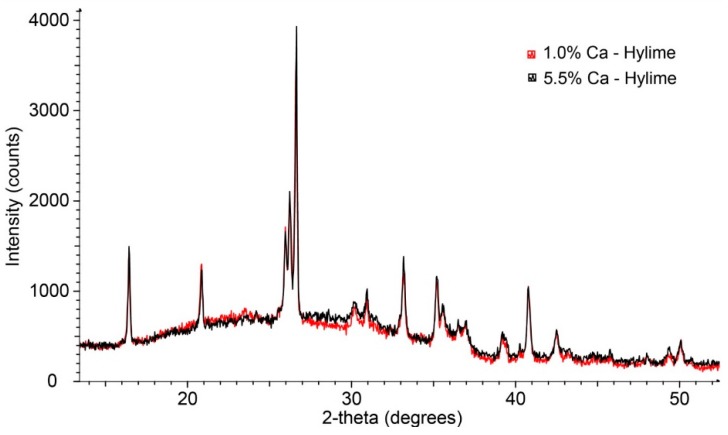
A selected portion of the XRD patterns from Muja fly ash/Bayer liquor geopolymer with 1.0 and 5 wt % addition of Ca(OH)_2_.

Figure 8 shows the XRD patterns collected from 1.0 wt % and 5.5 wt % calcium addition from Ca(OH)_2_ to Muja fly ash based geopolymers. There is a marginal increase in the amorphous geopolymer component for the 5.5 wt % sample, recognisable from the broad elevation in the pattern centred at ~28° 2θ (Figure 8). It is clear that no new phases have been formed by adding the Ca(OH)_2_ at either 1 or 5.5 wt % and curing at ambient temperature. It is apparent that the presence of Ca in samples cured at ambient temperature facilitates geopolymerisation leading to a strength increase [41].

It is apparent that addition of calcium to the formulation of Bayer derived fly ash geopolymer results in ambient temperature curing with increasing calcium resulting in higher compressive strength. The target compressive strength of 20 MPa is achieved with there being no discernible changes to the formation of geopolymer.

## 4. Conclusions

For a geopolymer concrete binding agent to be suitable for the replacement of Ordinary Portland Cement, the ability to cure at ambient temperature is considered essential to maximise the market opportunities. The novel approach of using Bayer process liquor as the activating solution for the manufacturing of geopolymers has opened the possibility of industrial scale synergy with significant reductions in product embodied energy. This paper has demonstrated that Bayer derived fly ash geopolymer formulations can be modified to achieve ambient cure.

Calcium was added to formulations utilising Collie or Muja power station fly ash to manufacture ambient temperature cured, Bayer derived geopolymer, achieving a minimum 20 MPa compressive strength.

Geopolymers made from Muja fly ash required less calcium addition yet resulted in a higher compressive strength than those made from Collie fly ash. This is believed to be a surface area effect of the fly ash resulting in greater reactivity.

The availability of calcium to the geopolymer appears critical to the product compressive strength. This would indicate that well defined calcium reagents are required for quality control and quality assurance.

The use of multiple industrial by-products to manufacture ambient curing geopolymers is a major step forward towards introducing commercially viable alternatives to Ordinary Portland Cement.

## Figures and Tables

**Table 1:** Composition of Bayer liquors used to synthesise geopolymers.

Source of Bayer Liquor	Al_2_O_3_ (g/L)	NaOH (g/L)
Kwinana	252.7	438.5
Wagerup	228.4	449.1

**Table 2:** Bulk composition (XRF) of fly ash.

Oxide	Collie	Muja
	wt %	wt %
SiO_2_	49.9 (2)	44.9 (2)
Al_2_O_3_	24.8 (2)	32.5 (2)
Fe_2_O_3_	16.60 (4)	10.6 (4)
CaO	1.8 (1)	2.0 (1)
K_2_O	0.61 (8)	0.69 (8)
TiO_2_	1.36 (2)	1.67 (3)
MgO	1.31 (6)	1.44 (6)
Na_2_O	0.4 (1)	0.6 (1)
P_2_O_5_	1.52 (4)	1.52 (4)
SrO	0.33 (1)	0.22 (1)
BaO	0.45 (1)	0.53 (1)
Other (includes LOI)	0.7 (1)	3.33 (3)
Si/Al (molar ratio)	1.71 (1)	1.17 (1)

Values in parentheses in this and all further tables correspond to the least significant figure in the estimated standard deviation to the left, all in weight percent. LOI is loss on ignition.

**Table 3:** The phase composition of the fly ash by Rietveld quantitative analysis.

Mineral/Phase	Collie	Muja
	wt %	wt %
Primary Quartz	13 (1)	5.9 (6)
Secondary Quartz	11.0 (2)	4.9 (3)
Hematite	2.42 (8)	1.13 (7)
Magnetite	2.0 (1)	3.4 (3)
Mullite	14 (1)	28 (1)
Maghemite C	6.6 (3)	–
Amorphous	51 (1)	57 (1)

**Table 4:** Composition of amorphous component of the fly ash.

Oxide	Collie	Muja
	wt %	wt %
SiO_2_	21 (2)	26 (2)
Al_2_O_3_	15 (1)	12 (1)
sum of aluminosilicates	36 (3)	38.1 (4)
Si/Al (molar ratio)	1.2	1.8

**Table 5:** Particle size and surface area of the fly ash and silica fume. Particle size (d) is presented as the cumulative volume diameter and the number in brackets represents the cut off value in percent.

Fly Ash/Fume	Surface Area (m^2^/cc)	d (10) (µm)	d (50) (µm)	d (80) (µm)	d (90) (µm)
Collie	1.01	2.7	16.0	49.3	81.9
Muja	2.24	1.1	4.4	13.2	24.6
Silica Fume (SF98)	18	0.3	0.5	–	0.8
